# Uracil-tegafur vs fluorouracil as postoperative adjuvant chemotherapy in Stage II and III colon cancer: A nationwide cohort study and meta-analysis

**DOI:** 10.1097/MD.0000000000025756

**Published:** 2021-05-07

**Authors:** Po-Huang Chen, Yi-Ying Wu, Cho-Hao Lee, Chi-Hsiang Chung, Yu-Guang Chen, Tzu-Chuan Huang, Ren-Hua Yeh, Ping-Ying Chang, Ming-Shen Dai, Shiue-Wei Lai, Ching-Liang Ho, Jia-Hong Chen, Yeu-Chin Chen, Je-Ming Hu, Sung-Sen Yang, Wu-Chien Chien

**Affiliations:** a Department of General Medicine; b Department of Internal Medicine; c Department of Internal Medicine, Division of Hematology and Oncology Medicine, Tri-Service General Hospital; d School of Public Health; e Department of Medical Research, Tri-Service General Hospital, National Defense Medical Center; f Taiwanese Injury Prevention and Safety Promotion Association (TIPSPA), Taipei, Taiwan, ROC; g UCL Cancer Institute, University College London, UK; h Division of Colorectal Surgery, Department of Surgery, National Defense Medical Center; i Division of Nephrology, Department of Medicine, Tri-Service General Hospital; j Graduate Institute of Medical Sciences; k Graduate Institutes of Life Sciences, National Defense Medical Center, Taipei, Taiwan, ROC.

**Keywords:** adjuvant chemotherapy, colon cancer, fluorouracil, tegafur-uracil

## Abstract

We conducted a population-based cohort study enrolling patients with Stage II and III colon cancer receiving postoperative adjuvant chemotherapy with uracil and tegafur (UFT) or fluorouracil (5-FU) from the Taiwan National Health Insurance Research Database from 2000 to 2015. The outcomes of the current study were disease-free survival (DFS) and overall survival (OS). Hazard ratios (HRs) were calculated by multivariate Cox proportional hazard regression models. We compared our effectiveness results from the literature by meta-analysis, which provided the best evidence. Severe adverse events were compared in meta-analysis of reported clinical trials. In the nationwide cohort study, UFT (14,486 patients) showed DFS similar to postoperative adjuvant chemotherapy (adjusted HR 1.037; 95% confidence interval [CI] 0.954–1.126; *P* = .397) and OS (adjusted HR 0.964; 95% CI 0.891–1.041; *P* = .349) compared with the 5-FU (866 patients). Our meta-analysis confirmed the similarity of effectiveness and found the incidence of leucopaenia was statistically significantly reduced in UFT (risk ratio 0.12; 95% CI 0.02–0.67; *I*^*2*^ = 0%). Through our analysis, we have confirmed that UFT is a well-tolerated adjuvant therapy choice, and has similar treatment efficacy as 5-FU in terms of DFS and OS in patients with Stage II and III colon cancer.

## 1 Introduction

The worldwide incidence of colon cancer has been increasing rapidly in recent decades and is the third most common cancer. In Taiwan, colon cancer is the most common cancer in men and the second most common in women.^[1]^ Radical surgical resection followed by adjuvant chemotherapy is the standard treatment for patients with Stage II and III colon cancer, which also provides a drastic reduction in local recurrence rates and improves disease-free survival (DFS) and overall survival (OS).^[2]^

Since the early 1990s, chemotherapy with fluorouracil (5-FU) has been a curative therapeutic choice for postoperative adjuvant chemotherapy in Stage II and III colon cancer.^[3]^ According to the results from the MOSAIC trial,^[4]^ adding oxaliplatin to a regimen of 5-FU significantly improved DFS and OS in patients with Stage II and III colon cancer, which established that oxaliplatin-containing regimens had been adopted as standard adjuvant chemotherapy in most clinical guidelines since the 2000s.^[5,6]^ However, combined intravenous 5-FU and leucovorin (LV) remains a therapeutic regimen of choice, especially for elderly patients and patients who cannot tolerate the neurotoxicity of oxaliplatin.^[7]^ As an alternative, uracil and tegafur (UFT), an oral drug derived from 5-FU derivatives, has been used in metronomic therapy following an intravenous 5-FU/LV plus oxaliplatin regimen for patients with Stage III colon cancer,^[8]^ or as postoperative adjuvant chemotherapy in Stage II and III colon cancer.^[9,10]^

The oral form of chemotherapy is attractive in clinical practice because of its low-grade toxicity and its convenience in an outpatient clinic setting.^[11]^ Given the expected clinical benefits in terms of safety, convenience and lower treatment cost,^[12]^ a consensus has not been reached as to whether UFT can be equivalent in efficacy to intravenous 5-FU in the adjuvant treatment of Stage II and III colon cancer.

Treatment efficacy can be influenced by many clinical factors in the real world; as a result, nonrandomized controlled trial data still play a role.^[13]^ Some cohort studies have evaluated the effectiveness of oral UFT vs intravenous 5-FU. For example, Hu et al^[14]^ had reported that long-course oral UFT treatment improved survival and had the potential to replace short-course infusional 5-FU as the standard adjuvant chemotherapy for patients with low-risk Stage II colon cancer. However, a large-scale population-based, long-term, retrospective cohort study is still lacking to clarify the uncertainties concerning its long-term adjuvant effects. Both oral UFT and 5-FU were approved for adjuvant chemotherapy for patients with Stage II and III colon cancer by the Taiwan National Health Insurance (NHI); therefore, we conducted this population-based retrospective study to further confirm the adjuvant effectiveness of UFT. We also combined our results with data from the available literature in a meta-analysis to provide the best evidence. Furthermore, we conducted a meta-analysis comparing severe adverse events (SAEs) of UFT vs 5-FU treatment. The aim of this nationwide cohort study and meta-analysis was to compare the effectiveness of oral adjuvant chemotherapy UFT with intravenous 5-FU in terms of DFS and OS in patients with Stage II and III colon cancer.

## 2 Method

### 2.1 Nationwide retrospective cohort study

#### 2.1.1 Data sources

The data of this present study were obtained from the Taiwan National Health Insurance Research Database (NHIRD). Taiwan began an NHI program on March 1, 1995. NHI is a single-payer program administered by the government, to finance health care for all residents in Taiwan. Approximately 99% of the Taiwanese population was covered, and 97% of the hospitals and clinics had contracts with the NHI program in Taiwan.^[15]^ We analyzed a representative database of 2,000,000 individuals called the Longitudinal Heath Insurance Database (LHID), which was randomly sampled from all NHI beneficiaries containing the complete medical records of each person insured from 2000 to 2015 by the National Health Research Institute. The LHID provides detailed information in terms of International Classification of Diseases, Ninth Revision (ICD-9-CM). After receiving National Health Research Institute approval, we used the LHID to conduct our study. To protect patient privacy and data security, personal identification information was encrypted before releasing the research database from the NHIRD.

#### 2.1.2 Sampled participants and study design

Between January 2000 and December 2015, we used the primary discharge diagnosis of colon cancer (ICD-9-CM 153–154.1) to identify the patients. The index date was defined as the date of the colon cancer diagnosis. We retrieved all prescription data for patients undergoing operative therapy within 6 months after the diagnosis. Patients who were prescribed UFT within 6 months after operation comprised the UFT cohort and those who were prescribed 5-FU comprised the 5-FU cohort. The exclusion criteria were

1. the date of cancer diagnosis (ICD-9-CM140–239) prior to the index date;
2. patients with secondary malignancy (ICD-9-CM:196–198.9);
3. patients with benign neoplasm of the colon (ICD-9-CM:211.3, 211.4);
4. patients receiving bevacizumab, cetuximab, capecitabine, irinotecan or oxaliplatin;
5. patients receiving both UFT and 5-FU or neither.

**Figure 1:**
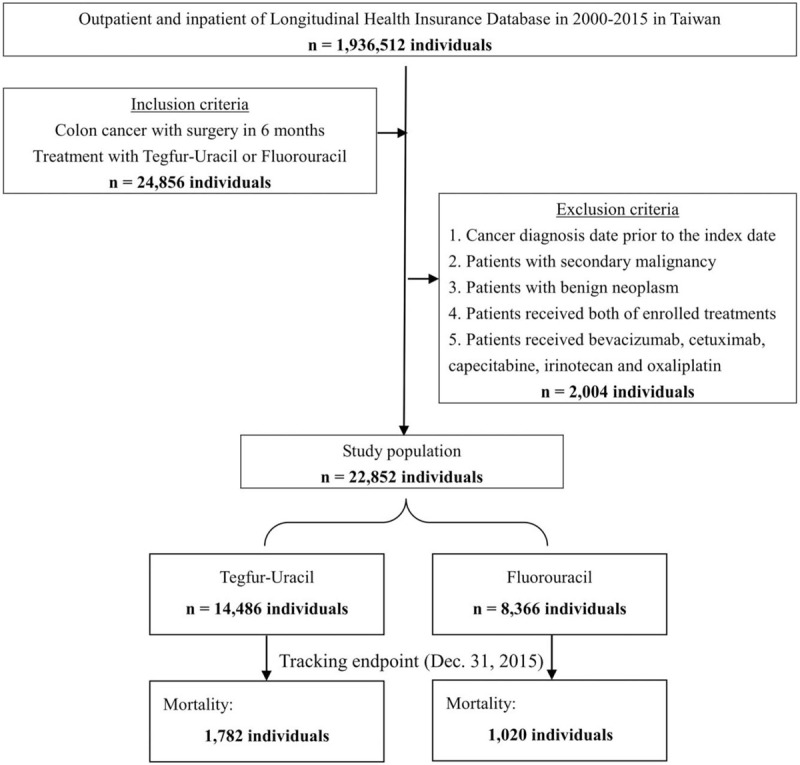
Flowchart of the study sample selection from the NHIRD in Taiwan. NHIRD = National Health Insurance Research Database.

The algorithm of this study is shown in Figure 1.

#### 2.1.3 Outcome, risk factors and comorbidities for cancers

The outcomes of the study were DFS and OS. The date of the colon cancer diagnosis was established as the index date for starting the measurement of follow-up person-years. DFS was defined as years from the index date to tumor recurrence (ICD9-CM 196.1–198.89, Supplementary Digital Content 1) or death. OS was defined as years from index date until death, withdrawal from the insurance system (mostly due to death) or until the end of 2015. Baseline comorbidities were identified: hypertension, diabetes mellitus (DM), chronic obstructive pulmonary disease (COPD), chronic kidney disease (CKD), ischemic heart disease (IHD), congestive heart disease (CHD), and stroke. The overall Charlson comorbidity index removed cancer (CCI_R) represented other miscellaneous comorbidities.^[16]^ The socioeconomic status of the study participants was approximated using insurance premium, level of care, and urbanization levels. Insurance premiums, which served as a proxy for income level, were grouped into 3 categories (≥ $35,000, $18,000–$34,999 and $1–$18,000 [New Taiwan dollars per month]). Based on information in the NHIRD, participants were also stratified by residence. Levels of care were stratified by the level of hospital judged by the Taiwanese government as central, regional or local hospital. Urbanization in Taiwan was categorized into 4 levels, with a lower level indicating a higher degree of urbanization.^[17]^

#### 2.1.4 Statistical analysis

The statistical software used was the Statistical Product and Service Solutions 23rd edition (SPSS; IBM Corp., Armonk, NY). The categorical variables were compared by Chi-Squared or Fisher exact test, and the continuous variables were compared by *t* test. We calculated the hazard ratios (HRs) and the 95% confidence interval (CI) using the Cox proportional hazards model to assess DFS and OS for UFT vs 5-FU, and a log-rank test was used to compare the differences between the cumulative survival curves. All baseline characteristics, including age, sex, insurance premium, level of care, urbanization, hypertension, DM, COPD, CKD, IHD, CHD, stroke, and CCI_R were adjusted when performing the multivariate Cox proportional hazards regression model. The *P* values of the log-rank test less than .05 were regarded as statistically significant.

#### 2.1.5 Ethics approval and consent to participate

Given the present study used de-identified secondary data, the patients were not directly involved in this study, and thus the need for consent was waived. This study was approved by the Tri-Service General Hospital Research Ethics Committee (TSGHIRB No. B-109–11).

### 2.2 Meta-analysis

The meta-analysis complied with a previously registered protocol for this with Open Science Framework (https://osf.io/kf84r) and the international register was developed by the Center for Open Science. The present study followed the Preferred Reporting Items for Systematic Reviews and Meta-analyses^[18]^ and the Meta-analysis of Observational Studies in Epidemiology guidelines.^[19]^

#### 2.2.1 Eligibility criteria and exclusion criteria

To be eligible for inclusion, studies had to report the findings from a randomized clinical trial or observational study investigating the efficacy or effectiveness of postoperative adjuvant chemotherapy with either UFT or 5-FU among individuals diagnosed with Stage II or III colon cancer. Studies were excluded if they were single-arm studies, conducted in patients with rectal cancer, conducted on a nonhuman (e.g., mice) or laboratory (e.g., cell line) population; focused on early-stage (Stage 0 or I) or metastatic (Stage IV) tumors; if DFS or OS outcomes were not reported; or examined a combination chemotherapy therapy (e.g., UFT or 5-FU combining with oxaliplatin or TS-1).

#### 2.2.2 Information sources and search strategy

We performed a comprehensive search without language restrictions, using PubMed, Embase, the Cochrane Central Register of Controlled Trials, and the Cochrane Database of Systematic Review. In order to ensure that no randomized controlled trials were missing, grey literature (conference abstract and doctoral thesis) was searched and reference lists of included articles were reviewed. The search was conducted on February, 2020. The detailed search strategy is described in the Supplementary Digital Content 2.

#### 2.2.3 Study quality

The quality of the randomized controlled trials was appraised by PHC and CHL, using the Cochrane Handbook for Systematic Reviews of Interventions.^[20]^ Furthermore, we assessed the quality of cohort studies using the Newcastle–Ottawa Scale.^[21]^ Any disagreement was resolved via group discussion. Risk-of-bias graphs were generated using Review Manager 5.3 software. (The Cochrane Collaboration, 2014; The Nordic Cochrane Centre, Copenhagen, Denmark).

#### 2.2.4 Outcome measurement and statistical analysis

The major outcomes of interest were DFS and OS, and the minor outcome was SAE (Grade 3/4 adverse events according to the Common Terminology Criteria of Adverse Events v4.03).^[22]^ Data are presented as HR and risk ratio (RR) with 95% CIs. To minimize bias from combining randomized and nonrandomized evidence, we used the design-adjusted analysis method, and we reduced the weight of nonrandomized studies.^[23]^ Given the expected between-study heterogeneity due to differences in patient populations, a random-effects DerSimonian–Laird model,^[24]^ using the reverse invariance method, was used to estimate the average measure of association. Heterogeneity was evaluated using the *I* squared (*I*^*2*^) statistic and Cochran Q test.^[25]^ Statistically significant heterogeneity was defined as *I*^*2*^ > 50% and for Cochran Q Test *P* < .1. Subgroup analyses were conducted by study types (RCT or cohort study) and whether the studies were performed in Eastern or Western countries in terms of the major outcomes. We evaluated whether treatment effects on both outcomes were robust by sensitivity analyses, which were performed based on the specific features of the study design to explore the impact of excluding our NHIRD analysis and excluding high risk-of-bias studies. The presence of publication bias was assessed via funnel plots and with Egger test.^[26]^ All statistical analyses were performed using the “metafor” and “meta” packages of R software. Statistical significance was defined at the 0.05 threshold, and all statistical tests were 2-sided.

## 3 Results

### 3.1 Nationwide retrospective cohort study

#### 3.1.1 Characteristics of study participants

A total of 22,852 eligible patients were enrolled in the study, and we excluded patients receiving both UFT and 5-FU or neither. Table 1 compares the characteristics and baseline comorbidity status between the UFT (n = 14,486) and 5-FU (n = 8366) cohorts. The percentage of male sex for the UFT and 5-FU cohorts was 59.99% and 60.53%, respectively. The mean ± SD age for the UFT and 5-FU cohorts was 66.75 ± 11.67 and 66.74 ± 11.69 years, respectively. The mean years of follow-up (± SD) in the UFT and 5-FU group were 4.10 ± 3.75 and 4.02 ± 3.67 years, respectively. There were no significant differences between the groups in terms of basic characteristics, such as sex, age, insured premium, urbanization and hospital level, or comorbidities, including hypertension, DM, COPD, CKD, IHD, CHD, stroke, or CCI_R index. (Social economic data was available: Supplementary Digital Content 3; Characteristics of study at the end of follow-up was available: Supplementary Digital Content 4).

**Table 1:** Characteristics of study in the baseline.

	Total		UFT		5-FU		
Treatment variables	n	%	n	%	n	%	*P value*
Total	22,852		14,486	63.39	8366	36.61	
Gender							.420
Male	13,754	60.19	8690	59.99	5064	60.53	
Female	9098	39.81	5796	40.01	3302	39.47	
Age (years ± SD)	66.75 ± 11.68	66.75 ± 11.67	66.74 ± 11.69	0.950			
HTN							
With	6588	28.83	4179	28.85	2409	28.80	.932
Without	16,264	71.17	10,307	71.15	5,957	71.20	
DM							
With	3897	17.05	2488	17.18	1409	16.84	.519
Without	18,955	82.95	11,998	82.82	6957	83.16	
COPD							
With	914	4.00	590	4.07	324	3.87	.457
Without	21,938	96.00	13,896	95.93	8042	96.13	
CKD							
With	282	1.23	177	1.22	105	1.26	.827
Without	22,570	98.77	14,309	98.78	8261	98.74	
IHD							
With	1196	5.23	757	5.23	439	5.25	.943
Without	21,656	94.77	13,729	94.77	7927	94.75	
CHD							
With	439	1.92	268	1.85	171	2.04	.304
Without	22,413	98.08	14,218	98.15	8195	97.96	
Stroke							
With	684	2.99	428	2.95	256	3.06	.652
Without	22,168	97.01	14,058	97.05	8110	96.94	
CCI_R	0.03 ± 0.24	0.03 ± 0.24	0.03 ± 0.23	0.999			

*P value*: categorical variables: Chi-Squared/Fisher exact test, continuous variables: *t* test. 5-FU = 5-Flurouracil, CCI_R = Charlson comorbidity index removed cancer, CHD = congestive heart disease, CKD = chronic kidney disease, COPD = chronic obstructive pulmonary disease, DM = diabetes mellitus, HTN = hypertension, IHD = ischemic heart disease, UFT = uracil-tegafur.

#### 3.1.2 Disease-free survival

**Figure 2:**
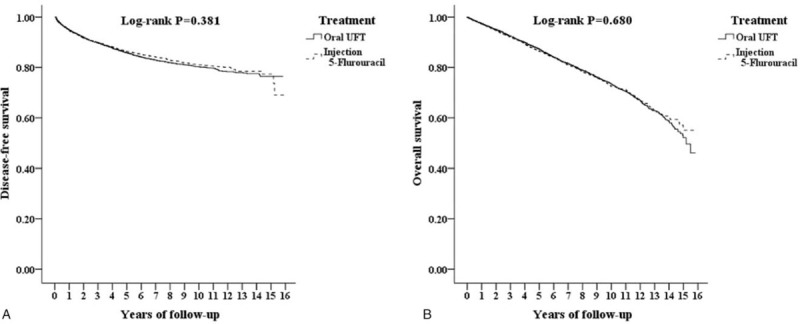
Kaplan–Meier for cumulative risk of disease-free survival (DFS, 2A) and overall survival (OS, 2B) among patients with Stage II and III colon cancer (NHIRD analysis).

A Cox proportional hazards model revealed no significant difference in DFS between the UFT and 5-FU cohorts (*P* for log-rank test = .381, Fig. 2A). All baseline characteristics, including age, sex, insurance premium, level of care, urbanization, hypertension, DM, COPD, CKD, IHD, CHD, stroke, and CCI_R were adjusted when performing a multivariate Cox proportional hazards regression model. The multivariate Cox proportional regression model indicated that, for patients receiving postoperative adjuvant chemotherapy in Stage II and III colon cancer compared with the 5-FU cohort, UFT was similar in DFS (adjusted HR 1.037; 95% CI 0.954–1.126; *P* = .397). Male sex, age older than 60 years, having comorbidities and the influence of CCI_R score were the important statically significant factors for shorter DFS.

#### 3.1.3 Overall survival

The Cox proportional hazards model revealed no significant difference in the cumulative OS between the UFT and 5-FU cohorts (*P* for log-rank test = .680, Fig. 2B). The multivariate Cox proportional regression model indicated that, compared with the 5-FU cohort, UFT was similar in OS (adjusted HR = 0.964; 95% CI 0.891–1.041; *P* = .349). Male sex, age older than 40 years, having comorbidities and the influence of CCI_R score were the significant factors for shorter OS. Central hospitals had lower mortality compared with local hospitals. (Supplementary Digital Content 5).

### 3.2 Meta-analysis

A flow diagram of the inclusion of studies is shown in Supplementary Digital Content-6. Our database search identified 402 publications after the removal of duplicates. After the screening process, 27 articles were reviewed in full, from which 4 studies (2 randomized trials and 2 observational studies) were deemed eligible for inclusion.

In total, 2537 patients were included from the 4 studies.^[14,27–29]^ All the studies examined DFS and OS, comparing oral UFT to intravenous 5-FU as postoperative adjuvant chemotherapy in carcinoma of the colon with Stage II disease, Stage III disease, or both. Descriptive characteristics are listed in Supplementary Digital Content 7, and the results from the study quality assessment are listed in Supplementary Digital Content 8.

**Figure 3:**
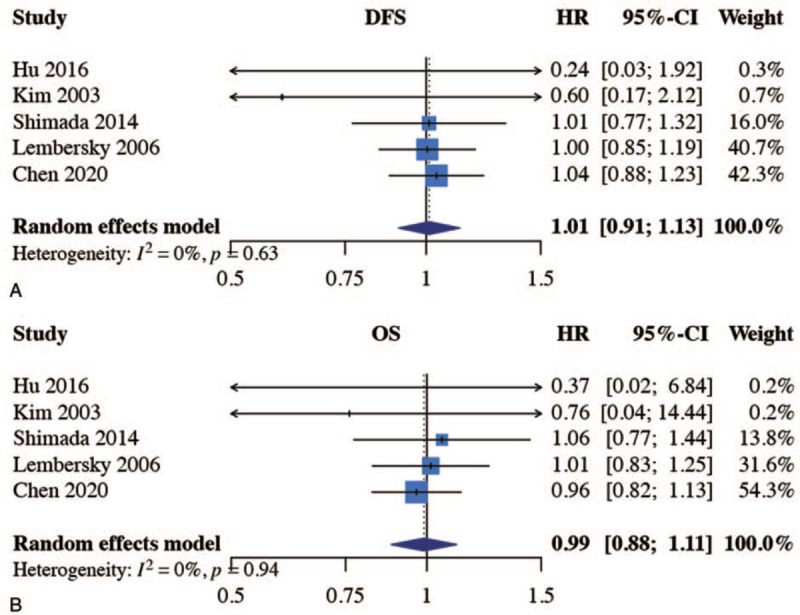
Meta-analysis of the efficacy for UFT vs 5-FU as postoperative adjuvant chemotherapy in stage II and III colon cancer: disease-free survival (DFS, 3A) and overall survival (OS, 3B). Outcome analyses were performed using hazard ratio (HR) with related 95% confidence intervals. CI = confidence interval, DFS = disease-free survival, HR = hazard ratio, OS = overall survival.

Combining our NHIRD analysis with the 2 randomized trials and the 3 observational studies enrolled, the results from the DFS and OS meta-analysis are summarized in a forest plot. For both outcomes, our analysis revealed that UFT and 5-FU did not differ with the random-effects model; the HR for DFS was 1.01 (95% CI 0.91–1.13, Fig. 3A) and the HR for OS was 0.99 (95% CI 0.88–1.11, Fig. 3B). There was no heterogeneity within this evidence base for the outcome of DFS (*I*^*2*^ = 0%, Cochran Q *P* = .63) or OS (*I*^*2*^ = 0%, Cochran Q *P* = .94). In the subgroup analysis, there was no significant difference between the UFT and 5-FU groups regarding the DFS and OS outcomes (Supplementary Digital Content 9). We also performed a sensitivity analysis, altering the choice of studies to remove our nationwide cohort study or high risk-of-bias studies, and the results did no change substantially (Supplementary Digital Content 10). Funnel plot and Egger test were not performed, because the studies included in the meta-analysis were fewer than 10; thus, the power of the tests was too low to distinguish the potential role of publication bias.^[30]^

We pooled data from 3 articles^[27–29]^ regarding SAE, involving 3834 patients, comparing UFT with 5-FU treatment as postoperative adjuvant chemotherapy in Stage II and III colon cancer. The incidence of leucopaenia was statistically significantly lower in the UFT cohort (RR 0.12; 95% CI 0.02–0.67; *I*^*2*^ = 0%). Compared with the 5-FU cohort, UFT treatment was reported to have similar incidence of anemia (2 trials^[27,29]^), diarrhea (3 trials^[27–29]^), nausea (3 trials^[27–29]^), and vomiting (3 trials^[27–29]^) (Table 2).

**Table 2:** Summary of meta-analysis comparing severe adverse events with UFT to 5-FU.

Comparison	Trials (n)	Severe adverse events	Summary estimate with 95% CI	I^2^ (%)	Cochran Q *P* value
UFT vs. 5-FU for postoperative adjuvant chemotherapy in stage II and III colon cancer	3 (3834)	Leucopaenia	RR: 0.12 [0.02; 0.67]^∗^	0%	.79
	2 (2301)	Anemia	RR: 1.63 [0.51; 5.25]	0%	.68
	3 (3834)	Diarrhea	RR: 1.01 [0.83; 1.24]	16%	.30
	3 (3834)	Nausea	RR: 0.99 [0.72; 1.35]	0%	.95
	3 (3834)	Vomiting	RR: 0.68 [0.46; 1.002]	0%	.72

5-FU = 5-Flurouracil, CI = confidence interval, RR = risk ratio, UFT = uracil-tegafur. Values of RR less than 1 indicate a reduction in risk for the events of severe adverse effect with the UFT group.

∗ Represents statistically significant outcome estimate; Statistically significant heterogeneity was defined as *I*^*2*^ > 50% and Cochran Q *P* value < .1.

## 4 Discussion

To the best of our knowledge, ours represents the largest real-world study to date to evaluate the effectiveness of UFT as adjuvant chemotherapy after surgical resection in patients with Stage II and III colon cancer. This nationwide, population-based, retrospective cohort study reveals that UFT presented similar effectiveness to 5-FU with respect to DFS and OS in a 15-year follow-up. Furthermore, we confirmed treatment efficacy by a meta-analysis of survival between the UFT and 5-FU groups by using data from recently published articles and the cases in this study. The results between the nationwide cohort study and the meta-analysis were consistent, which confirmed that the treatment effect of oral UFT was similar to intravenous 5-FU. The meta-analysis also found an advantage in the UFT cohort for reducing leucopaenic events.

Oral 5-FU derivatives are preferred because of their convenience, which has led to the development of several oral 5-FU derivatives with various properties. Since 1997, the weekly 24-hour infusion of high-dose 5-FU and leucovorin (LV) was reported by Yeh et al in Taiwan,^[31]^ this regimen became widely using and common therapeutic chemotherapy nationwide.^[32]^ The usual dosage would be weekly 24-hour infusion of a maximal tolerable dose of 5-FU (2600 mg/m^2^) and LV (500 mg/m^2^), a weekly 1-day course for a period of 6 months.

In a multicenter retrospective cohort study, UFT is commonly used in Japan for patients with Stage III colon cancer, especially in older patients.^[33]^ The composition of UFT is 1-(2-tetrahydrofuryl)-5-FU (tegafur) and uracil in a molar ratio of 1:4. Tegafur is converted to 5-FU in vivo, acting as a prodrug to 5-FU. Co-administration of uracil enhances the concentration of 5-FU in tumors and the resulting antitumor activity of tegafur.^[34]^ UFT has been demonstrated to have pharmacokinetics comparable to 5-FU in both Japanese and American patients.^[35]^ Oral UFT capsule contains of tegafur 100 mg and uracil 224 mg, administered with 2 capsules twice per day, equivalent to 400 mg of tegafur a day in Taiwan.^[36]^ The duration depended on the physician's clinical decision or the patient's condition. The Taiwan NHI reimburses the medical cost of UFT for 2 years at most.

Regarding our meta-analysis results for SAE, UFT represents another option, given it reduced the neutropenia rate compared with 5-FU. The safety analysis of the phase III trial, JCOG0205,^[29]^ revealed that the UFT/LV group had fewer neutropenic events. In a phase II study^[37]^ evaluating the toxicity profile of oral UFT in Chinese patients with metastatic colorectal cancer, Lin et al had revealed that the most frequently observed adverse effects were nausea and diarrhea. Furthermore, the myelosuppression event was mild, resulting in no episode of febrile neutropenia and less documented infection. UFT administration had fewer hand-foot syndromes,^[35]^ which can seriously impair quality of life in other oral chemotherapy regimens, such as capecitabine.^[38]^

Given the usage of oral UFT or intravenous 5-FU is typical in clinical practice, the cost-effectiveness evaluation is important for understanding the economic impact of these alternative regimens. In Taiwan, the NHI reimburses the medical cost of UFT and 5-FU for postoperative adjuvant chemotherapy Stages II and III colorectal cancer. Hsu et al^[12]^ had conducted a cost minimization comparison analysis of oral UFT/LV vs 5-FU/LV, and reflected a total cost saving per patient of US$3709.16–$5094.77 for UFT, along with 72 hours less time spent per patient. The analysis concluded that oral UFT/LV treatment has advantages in cost- and time-effectiveness. In a Borner et al^[39]^ study, 84% of the patients preferred an oral UFT regimen over intravenous therapies if the efficacy were similar. The UFT regimen showed the greater convenience of home treatment, given it could be administered orally via capsules. Patients do not need to travel daily or weekly to hospital, and there was better compliance among the participating patients.^[11]^

There are several limitations that should be taken into consideration when interpreting the results. First, the NHIRD lacked data on specific potentially important information, such as the severity of lymph node involvement. Second, our study relied on claims data and diagnostic codes to identify patients with cancer, which might lead to disease misclassification. Third, we could not investigate the cause of death, the adverse events of adjuvant chemotherapy, or the quality of surgery, due to the limitations of NHIRD. Furthermore, the data had insufficiently detailed clinical information, such as microsatellite instability, body mass index, physical activity, family history of cancer, and disease stage or status, as well as the reasons for each patient's treatment plan, for which is an intrinsic unavailability of data. As a result, there might be unmeasured residual confounding factors in the analysis that could have biased the results estimated in this study. Further studies accessing and examining the data from individual participants should allow for further identification and control of potential confounding factors.

Despite the limitations, our population-based cohort study provides powerful and generalizable real-world experience with a very large database,^[40]^ strengthening the statistical power for the effectiveness between UFT and 5-FU. The patients presented a wide range of demographic characteristics, enabling us to perform stratified analyses by age, sex, socioeconomic status, and comorbidities, without losing precision. Our meta-analysis provides a more comprehensive and precise estimate of the effectiveness, concurs with the current available evidence, and confirms the similar effectiveness of UFT in survival outcomes.

In conclusion, our nationwide cohort study and meta-analysis revealed that UFT treatment as adjuvant chemotherapy has effectiveness similar to 5-FU in DFS and OS, is well-tolerated, and reduces the incidence of leucopaenia in patients with postoperative Stage II and III colon cancer.

## Acknowledgments

The authors would like to thank Enago (www.enago.tw) for the English language review and Hong-Jie Jhou MD for the figure editing.

## Author contributions

**Conceptualization:** Po-Huang Chen, Yi-Ying Wu, Cho-Hao Lee, Yu-Guang Chen, Je-Ming Hu.

**Formal analysis:** Cho-Hao Lee, Chi-Hsiang Chung, Wu-Chien Chien.

**Funding acquisition:** Wu-Chien Chien.

**Investigation:** Ren-Hua Yeh, Ping-Ying Chang, Ming-Shen Dai, Shiue-Wei Lai, Jia-Hong Chen, Yeu-Chin Chen.

**Methodology:** Po-Huang Chen, Chi-Hsiang Chung, Wu-Chien Chien.

**Supervision:** Ching-Liang Ho.

**Writing – original draft:** Po-Huang Chen, Yi-Ying Wu.

**Writing – review & editing:** Yu-Guang Chen, Tzu-Chuan Huang, Sung-Sen Yang.

## Supplementary Material

Supplemental Digital Content

## Supplementary Material

Supplemental Digital Content

## Supplementary Material

Supplemental Digital Content

## Supplementary Material

Supplemental Digital Content

## Supplementary Material

Supplemental Digital Content

## Supplementary Material

Supplemental Digital Content

## Supplementary Material

Supplemental Digital Content

## Supplementary Material

Supplemental Digital Content

## Supplementary Material

Supplemental Digital Content

## Supplementary Material

Supplemental Digital Content

## References

[1] Kuo C-N Liao Y-M Kuo L-N. Cancers in Taiwan: practical insight from epidemiology, treatments, biomarkers, and cost. J Formos Med Assoc 2019;119:1731–41. 31522970 10.1016/j.jfma.2019.08.023

[2] Benson AB 3rd Schrag D Somerfield MR. American Society of Clinical Oncology recommendations on adjuvant chemotherapy for stage II colon cancer. J Clin Oncol 2004;22:3408–19. 15199089 10.1200/JCO.2004.05.063

[3] Wolmark N Rockette H Mamounas E. Clinical trial to assess the relative efficacy of fluorouracil and leucovorin, fluorouracil and levamisole, and fluorouracil, leucovorin, and levamisole in patients with Dukes’ B and C carcinoma of the colon: results from National Surgical Adjuvant Breast and Bowel Project C-04. J Clin Oncol 1999;17:3553–9. 10550154 10.1200/JCO.1999.17.11.3553

[4] André T Boni C Navarro M. Improved overall survival with oxaliplatin, fluorouracil, and leucovorin as adjuvant treatment in Stage II or III colon cancer in the MOSAIC trial. J Clin Oncol 2009;27:3109–16. 19451431 10.1200/JCO.2008.20.6771

[5] Al BB Alan PV Mahmoud MA-H. NCCN guidelines insights: colon cancer, Version 2.2018. J Natl Compr Canc Netw 2018;16:359–69. 29632055 10.6004/jnccn.2018.0021 PMC10184502

[6] Hashiguchi Y Muro K Saito Y. Japanese Society for Cancer of the Colon and Rectum (JSCCR) guidelines 2019 for the treatment of colorectal cancer. Int J Clin Oncol 2020;25:01–42. 10.1007/s10147-019-01485-z PMC6946738 31203527

[7] Argyriou AA. Updates on oxaliplatin-induced peripheral neurotoxicity (OXAIPN). Toxics 2015;3:187–97. 29056657 10.3390/toxics3020187 PMC5634688

[8] Huang WY Ho CL Lee CC. Oral tegafur-uracil as metronomic therapy following intravenous FOLFOX for stage III colon cancer. PLoS One 2017;12:e0174280. 28328969 10.1371/journal.pone.0174280 PMC5362219

[9] Sakamoto J Hamada C Yoshida S. An individual patient data meta-analysis of adjuvant therapy with uracil-tegafur (UFT) in patients with curatively resected rectal cancer. Br J Cancer 2007;96:1170–7. 17375049 10.1038/sj.bjc.6603686 PMC2360162

[10] Hamaguchi T Shirao K Moriya Y. Final results of randomized trials by the National Surgical Adjuvant Study of Colorectal Cancer (NSAS-CC). Cancer Chemother Pharmacol 2011;67:587–96. 20490797 10.1007/s00280-010-1358-1

[11] Meguro M Furuhata T Okita K. Clinical compliance with an oral uracil/tegafur (UFT) plus leucovorin (LV) regimen as adjuvant chemotherapy in Japanese colorectal cancer patients. Int J Clin Oncol 2009;14:402–7. 19856047 10.1007/s10147-009-0888-1

[12] Hsu TC Wang CC. Cost minimization comparison of oral UFT/leucovorin vs 5-fluorouracil/leucovorin as adjuvant therapy for colorectal cancer in Taiwan. J Comp Eff Res 2019;8:73–9. 30560687 10.2217/cer-2018-0078

[13] Lin LY Warren-Gash C Smeeth L. Data resource profile: the National Health Insurance Research Database (NHIRD). Epidemiol Health 2018;40:e2018062. 30727703 10.4178/epih.e2018062 PMC6367203

[14] Hu JM Chou YC Wu CC. Adjuvant chemotherapy with tegafur/uracil for more than 1 year improves disease-free survival for low-risk Stage II colon cancer. J Chin Med Assoc 2016;79:477–88. 27329402 10.1016/j.jcma.2016.04.001

[15] Wu T-Y Majeed A Kuo KN. An overview of the healthcare system in Taiwan. London J Prim Care (Abingdon) 2010;3:115–9. 10.1080/17571472.2010.11493315 PMC3960712 25949636

[16] Charlson ME Pompei P Ales KL. A new method of classifying prognostic comorbidity in longitudinal studies: development and validation. J Chronic Dis 1987;40:373–83. 3558716 10.1016/0021-9681(87)90171-8

[17] Huang NC Kung SF Hu SC. The Relationship between urbanization, the built environment, and physical activity among older adults in Taiwan. Int J Environ Res Public Health 2018;15: doi:10.3390/ijerph15050836. 10.3390/ijerph15050836 PMC5981875 29695078

[18] Moher D Liberati A Tetzlaff J. Preferred reporting items for systematic reviews and meta-analyses: the PRISMA statement. BMJ 2009;339:b2535. 19622551 10.1136/bmj.b2535 PMC2714657

[19] Stroup DF Berlin JA Morton SC. Meta-analysis of observational studies in epidemiology: a proposal for reporting. Meta-analysis of observational studies in epidemiology (MOOSE) group. JAMA 2000;283:2008–12. 10789670 10.1001/jama.283.15.2008

[20] Higgins JPTGS. Cochrane Handbook for Systematic Reviews of Interventions Version 5.1.0 [updated March 2011]. 2011;The Cochrane Collaboration, United Kingdom

[21] Kim SY Park JE Lee YJ. Testing a tool for assessing the risk of bias for nonrandomized studies showed moderate reliability and promising validity. J Clin Epidemiol 2013;66:408–14. 23337781 10.1016/j.jclinepi.2012.09.016

[22] Zhang S Liang F Tannock I. Use and misuse of common terminology criteria for adverse events in cancer clinical trials. BMC Canc 2016;16:392. 10.1186/s12885-016-2408-9 PMC4932726 27377548

[23] Efthimiou O Mavridis D Debray TP. Combining randomized and non-randomized evidence in network meta-analysis. Stat Med 2017;36:1210–26. 28083901 10.1002/sim.7223

[24] DerSimonian R Laird N. Meta-analysis in clinical trials revisited. Contemp Clin Trials 2015;45 (Pt A):139–45. 26343745 10.1016/j.cct.2015.09.002 PMC4639420

[25] Higgins JPT Thompson SG Deeks JJ. Measuring inconsistency in meta-analyses. BMJ 2003;327:557–60. 12958120 10.1136/bmj.327.7414.557 PMC192859

[26] Egger M Smith GD Schneider M. Bias in meta-analysis detected by a simple, graphical test. BMJ 1997;315:629–34. 9310563 10.1136/bmj.315.7109.629 PMC2127453

[27] Kim DJ Kim TI Suh JH. Oral tegafur-uracil plus folinic acid vs intravenous 5-fluorouracil plus folinic acid as adjuvant chemotherapy of colon cancer. Yonsei Med J 2003;44:665–75. 12950123 10.3349/ymj.2003.44.4.665

[28] Lembersky BC Wieand HS Petrelli NJ. Oral uracil and tegafur plus leucovorin compared with intravenous fluorouracil and leucovorin in stage II and III carcinoma of the colon: results from National Surgical Adjuvant Breast and Bowel Project Protocol C-06. J Clin Oncol 2006;24:2059–64. 16648506 10.1200/JCO.2005.04.7498

[29] Shimada Y Hamaguchi T Mizusawa J. Randomised phase III trial of adjuvant chemotherapy with oral uracil and tegafur plus leucovorin vs intravenous fluorouracil and levofolinate in patients with stage III colorectal cancer who have undergone Japanese D2/D3 lymph node dissection: final results of JCOG0205. Eur J Cancer 2014;50:2231–40. 24958736 10.1016/j.ejca.2014.05.025

[30] Lau J Ioannidis JPA Terrin N. The case of the misleading funnel plot. BMJ 2006;333:597–600. 16974018 10.1136/bmj.333.7568.597 PMC1570006

[31] Yeh KH Cheng AL Lin MT. A phase II study of weekly 24-hour infusion of high-dose 5-fluorouracil and leucovorin (HDFL) in the treatment of recurrent or metastatic colorectal cancers. Anticancer Res 1997;17 (5b):3867–71. 9427794

[32] Chen L-T Whang-Peng J. Current status of clinical studies for colorectal cancer in Taiwan. Clin Colorectal Canc 2004;4:196–203. 10.3816/ccc.2004.n.020 15377403

[33] Kawamura H Morishima T Sato A. Effect of adjuvant chemotherapy on survival benefit in stage III colon cancer patients stratified by age: a Japanese real-world cohort study. BMC Cancer 2020;20:19. 31906959 10.1186/s12885-019-6508-1 PMC6945708

[34] Pazdur R Lassere Y Diaz-Canton E. Phase I trial of uracil-tegafur (UFT) plus oral leucovorin: 14-day schedule. Invest N Drugs 1997;15:123–8. 10.1023/a:1005808822565 9220291

[35] Shirao K Hoff PM Ohtsu A. Comparison of the efficacy, toxicity, and pharmacokinetics of a uracil/tegafur (UFT) plus oral leucovorin (LV) regimen between Japanese and American patients with advanced colorectal cancer: joint United States and Japan study of UFT/LV. J Clin Oncol 2004;22:3466–74. 15277535 10.1200/JCO.2004.05.017

[36] Chen T-C Jeng Y-M Liang J-T. Metronomic chemotherapy with tegafur-uracil following radical resection in stage II colorectal cancer. J Formos Med Assoc 2020. 10.1016/j.jfma.2020.09.014 33023787

[37] Lin J-K Wang W-S Hsieh R-K. Phase II study of oral tegafur-uracil and folinic acid as first-line therapy for metastatic colorectal cancer: Taiwan experience. Japanese J Clin Oncol 2000;30:510–4. 10.1093/jjco/hyd124 11155922

[38] Inokuchi M Ishikawa S Furukawa H. Treatment of capecitabine-induced hand-foot syndrome using a topical retinoid: a case report. Oncol Lett 2014;7:444–8. 24396465 10.3892/ol.2013.1706 PMC3881915

[39] Borner MM Schoffski P de Wit R. Patient preference and pharmacokinetics of oral modulated UFT vs intravenous fluorouracil and leucovorin: a randomised crossover trial in advanced colorectal cancer. Eur J Cancer 2002;38:349–58. 11818199 10.1016/s0959-8049(01)00371-9

[40] Hsing AW Ioannidis JP. Nationwide population science: lessons from the Taiwan National Health Insurance Research Database. JAMA Intern Med 2015;175:1527–9. 26192815 10.1001/jamainternmed.2015.3540

