# Supplementary material for: Uracil-tegafur vs fluorouracil as postoperative adjuvant chemotherapy in Stage II and III colon cancer: A nationwide cohort study and meta-analysis
Source: Medicine (Baltimore). 2021 May 7;100(18):e25756. doi: 10.1097/MD.0000000000025756 (PMC8104207; doi:10.1097/MD.0000000000025756)
Supplement: Supplemental Digital Content [file medi-100-e25756-s001.pdf]

Supplementary Digital Content 1. ICD-9-CM, NHI code and definition

ICD-9-CM, NHI code and definition	
	**ICD-9-CM / NHI code / definition**
**Study population**	
Colorectal cancer	153-154.1
Surgery	OP45.21, OP45.71-OP45.76, OP45.79, OP45.8, OP48.4-OP48.6
**Excluding:** Other cancers	140-239, excluding colorectal cancer
**Treatment**	Within 6-months after colorectal cancer surgery
Oral	Uracil-tegafur
Injection	5-Fluorouracil
**Events:** Prognosis	
Recurrence	196.1-198.89
Mortality	
**Comorbidities**	
Hypertension (HTN)	401-405
Diabetes mellitus (DM)	250
Chronic obstructive pulmonary disease (COPD)	490-496
Chronic kidney disease (CKD)	585
Ischemic heart disease (IHD)	410-414
Congestive heart disease (CHD)	428-429
Stroke	430-438
**Charlson comorbidity index revised (**CCI_R)	CCI removed cancer, HTN, DM, COPD, CKD, IHD, CHD, and stroke
