# Supplementary material for: Uracil-tegafur vs fluorouracil as postoperative adjuvant chemotherapy in Stage II and III colon cancer: A nationwide cohort study and meta-analysis
Source: Medicine (Baltimore). 2021 May 7;100(18):e25756. doi: 10.1097/MD.0000000000025756 (PMC8104207; doi:10.1097/MD.0000000000025756)
Supplement: Supplemental Digital Content [file medi-100-e25756-s002.pdf]

Supplementary Digital Content 10. Sensitivity analysis of outcomes

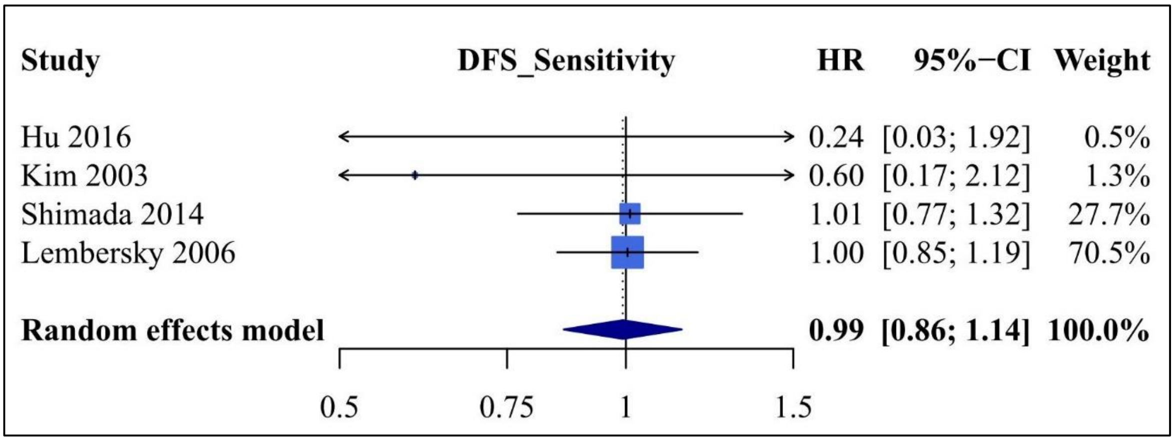

Sensitivity analysis of excluding NHIRD analysis in outcome of disease-free survival

DFS, disease-free survival; HR, hazard ration; CI, confidence interval; RCT, randomized control study

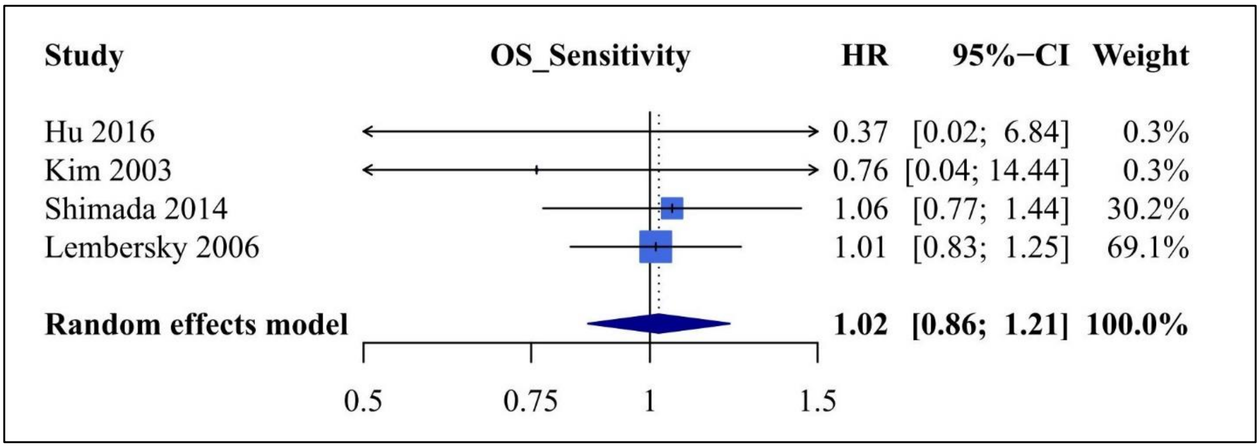

Sensitivity analysis of excluding NHIRD analysis in outcome of overall survival

OS, overall survival; HR, hazard ration; CI, confidence interval; RCT, randomized control study

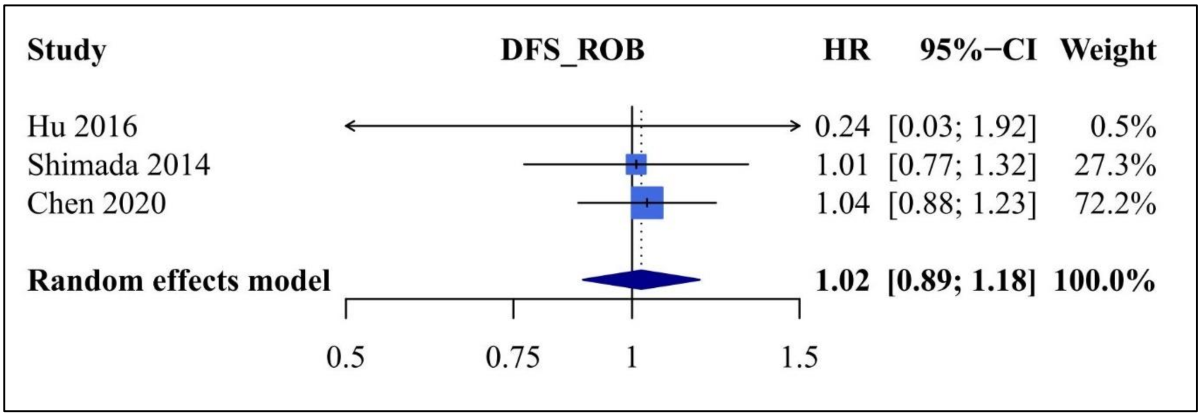

Sensitivity analysis of excluding high-risk of bias studies in outcome of disease-free survival

DFS, disease-free survival; HR, hazard ration; CI, confidence interval; RCT, randomized control study

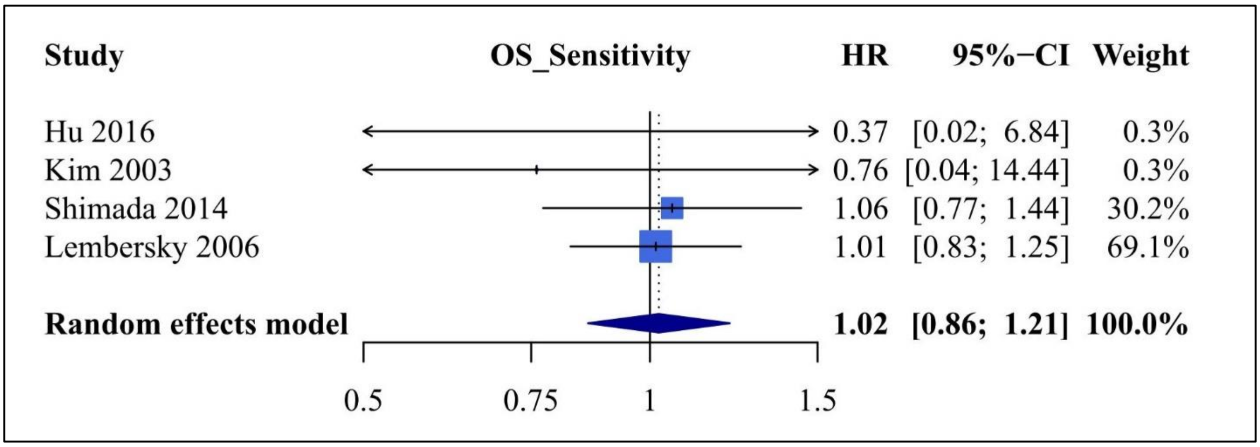

Sensitivity analysis of excluding high-risk of bias studies in outcome of overall survival

OS, overall survival; HR, hazard ration; CI, confidence interval; RCT, randomized control study
