# Supplementary material for: Uracil-tegafur vs fluorouracil as postoperative adjuvant chemotherapy in Stage II and III colon cancer: A nationwide cohort study and meta-analysis
Source: Medicine (Baltimore). 2021 May 7;100(18):e25756. doi: 10.1097/MD.0000000000025756 (PMC8104207; doi:10.1097/MD.0000000000025756)
Supplement: Supplemental Digital Content [file medi-100-e25756-s003.pdf]

Supplementary Digital Content 2. Search strategy

MEDLINE (Ovid) search strategy to February, 2020

1. (Colo* adj2 (cancer or tumour* or tumor* or neoplasm* or carcinoma* or adenocarcinoma*)).tw,kw
2. exp colorectal cancer/
3. ((Adjuvant or postoperative or post-operative or postsurgical or post-surgical or "after surger*") adj2 (therap* or treatment* or chemotherap*)).tw,kw.
4. 5-FU.tw,kw.
5. Fluorouracil.tw,kw.
6. Leucovorin.tw,kw.
7. UFT.tw,kw.
8. Tegufur.tw,kw.
9. uracil.tw,kw.
10. exp adjuvant chemotherapy/
11. (chemotherapy or therapy or treatment).tw,kw.
12. 1 or 2
13. 3 or 4 or 5 or 6 or 7 or 8 or 9 or 10 or 11
14. 12 and 13
15. limit 14 to (english language)
16. limit 15 to (conference abstract or conference paper or "conference review")

Embase search strategy to February, 2020

1. (Colo* adj2 (cancer or tumour* or tumor* or neoplasm* or carcinoma* or adenocarcinoma*)).tw,kw
2. exp colorectal cancer/
3. ((Adjuvant or postoperative or post-operative or postsurgical or post-surgical or "after surger*") adj2 (therap* or treatment* or chemotherap*)).tw,kw.
4. 5-FU.tw,kw.
5. Fluorouracil.tw,kw.
6. Leucovorin.tw,kw.
7. UFT.tw,kw.
8. Tegufur.tw,kw.
9. uracil.tw,kw.
10. exp adjuvant chemotherapy/
11. (chemotherapy or therapy or treatment).tw,kw.
12. 1 or 2
13. 3 or 4 or 5 or 6 or 7 or 8 or 9 or 10 or 11
14. 12 and 13
15. limit 14 to (english language)

16. limit 15 to (conference abstract or conference paper or "conference review")

Cochrane Central Register of Controlled Trials search strategy to February, 2020

("colonic neoplasms"[MeSH Terms] OR ("colonic"[All Fields] AND "neoplasms"[All Fields]) OR "colonic neoplasms"[All Fields] OR ("colon"[All Fields] AND "cancer"[All Fields]) OR "colon cancer"[All Fields]) AND ("UFT" [All Fields] OR "Tegufur"[All Fields] OR " uracil "[All Fields]) AND ("fluorouracil"[MeSH Terms] OR "fluorouracil"[All Fields])

Cochrane Database of Systematic Review search strategy to February, 2020

("colonic neoplasms"[MeSH Terms] OR ("colonic"[All Fields] AND "neoplasms"[All Fields]) OR "colonic neoplasms"[All Fields] OR ("colon"[All Fields] AND "cancer"[All Fields]) OR "colon cancer"[All Fields]) AND ("UFT" [All Fields] OR "Tegufur"[All Fields] OR " uracil "[All Fields]) AND ("fluorouracil"[MeSH Terms] OR "fluorouracil"[All Fields])
