# Supplementary material for: Uracil-tegafur vs fluorouracil as postoperative adjuvant chemotherapy in Stage II and III colon cancer: A nationwide cohort study and meta-analysis
Source: Medicine (Baltimore). 2021 May 7;100(18):e25756. doi: 10.1097/MD.0000000000025756 (PMC8104207; doi:10.1097/MD.0000000000025756)
Supplement: Supplemental Digital Content [file medi-100-e25756-s004.pdf]

Supplementary Digital Content 3. Characteristics of study in the baseline - Social economic data

Treatment Variables	Total		UFT		5-FU		*p-value*
	**n**	**%**	**n**	**%**	**n**	**%**	
**Total**	22,852		14,486	63.39	8,366	36.61	
**Insured premium (NT$)**							0.119
<18,000	22,450	98.24	14,250	98.37	8,200	98.02	
18,000-34,999	323	1.41	192	1.33	131	1.57	
≧35,000	79	0.35	44	0.30	35	0.42	
**Urbanization level**							0.817
1 (The highest)	8,870	38.81	5,641	38.94	3,229	38.60	
2	10,929	47.83	6,932	47.85	3,997	47.78	
3	1,112	4.87	700	4.83	412	4.92	
4 (The lowest)	1,941	8.49	1,213	8.37	728	8.70	
**Level of care**							0.934
Hospital center	12,689	55.53	8,046	55.54	4,643	55.50	
Regional hospital	8,646	37.83	5,485	37.86	3,161	37.78	
Local hospital	1,517	6.64	955	6.59	562	6.72	
***p-value*: categorical variables: chi-squared/Fisher's exact test; continuous variables: t-test**							

UFT, uracil-tegafur; 5-FU, 5-Flurouracil; HTN, hypertension; DM, diabetes mellitus; COPD, chronic obstructive pulmonary disease; CKD, chronic kidney disease; IHD, ischemic heart disease; CHD, congestive heart disease; CCI_R, Charlson comorbidity index removed cancer
