# Supplementary material for: Uracil-tegafur vs fluorouracil as postoperative adjuvant chemotherapy in Stage II and III colon cancer: A nationwide cohort study and meta-analysis
Source: Medicine (Baltimore). 2021 May 7;100(18):e25756. doi: 10.1097/MD.0000000000025756 (PMC8104207; doi:10.1097/MD.0000000000025756)
Supplement: Supplemental Digital Content [file medi-100-e25756-s005.pdf]

Supplementary Digital Content 4. Characteristics of study at the end of follow-up, and follow-up period

Treatment Variables	UFT		5-FU	
	n	%	n	%
**Total**	14,486	63.39	8,366	36.61
**Disease recurrence**				
Without	12,916	89.16	20,485	73.66
With	1,570	10.84	7,324	26.34
**Mortality**				
Without	12,704	87.70	23,520	84.58
With	1,782	12.30	4,289	15.42
**Gender**				
Male	8,690	59.99	5,064	60.53
Female	5,796	40.01	3,302	39.47
**Age (years ± SD)**	69.23 ± 14.22		69.29 ± 14.16	
**Insured premium (NT$)**				
<18,000	14,250	98.37	8,200	98.02
18,000-34,999	192	1.33	131	1.57
≧35,000	44	0.30	35	0.42
**HTN**				
Without	10,218	70.54	5,967	71.32
With	4,268	29.46	2,399	28.68
**DM**				
Without	11,611	80.15	6,679	79.84
With	2,875	19.85	1,687	20.16
**COPD**				
Without	13,541	93.48	7,826	93.55
With	945	6.52	540	6.45
**CKD**				
Without	13,679	94.43	7,928	94.76
With	807	5.57	438	5.24
**IHD**				
Without	12,996	89.71	7,544	90.17
With	1,490	10.29	822	9.83
**CHD**				

Without	13,789	95.19	7,960	95.15
With	697	4.81	406	4.85
**Stroke**				
Without	13,510	93.26	7,790	93.11
With	976	6.74	576	6.89
**CCI_R**	0.08 ± 0.47		0.07 ± 0.44	
**Urbanization level**				
1 (The highest)	4,691	32.38	2,678	32.01
2	6,731	46.47	3,874	46.31
3	978	6.75	562	6.72
4 (The lowest)	2,086	14.40	1,252	14.97
**Level of care**				
Hospital center	5,789	39.96	3,354	40.09
Regional hospital	6,316	43.60	3,595	42.97
Local hospital	2,381	16.44	1,417	16.94

UFT, uracil-tegafur; 5-FU, 5-Flurouracil; HTN, hypertension; DM, diabetes mellitus; COPD, chronic obstructive pulmonary disease; CKD, chronic kidney disease; IHD, ischemic heart disease; CHD, congestive heart disease; CCI_R, Charlson comorbidity index removed cancer

**Years of follow-up**				
**Treatment**	**Min**	**Median**	**Max**	**Mean ± SD**
Oral uracil-tegafur	0.01	3.17	15.84	4.10 ± 3.75
Injection 5-Flurouracil	0.01	3.07	15.92	4.02 ± 3.67
