# Supplementary material for: Uracil-tegafur vs fluorouracil as postoperative adjuvant chemotherapy in Stage II and III colon cancer: A nationwide cohort study and meta-analysis
Source: Medicine (Baltimore). 2021 May 7;100(18):e25756. doi: 10.1097/MD.0000000000025756 (PMC8104207; doi:10.1097/MD.0000000000025756)
Supplement: Supplemental Digital Content [file medi-100-e25756-s006.pdf]

Supplementary Digital Content 5. Cox regression analysis of disease-free survival and overall survival in relation to patients' characteristics

	**Disease-free survival**				**Overall survival**			
**Variables**	**Adjusted HR**	**95% CI**	**95% CI**	***P***	**Adjusted HR**	**95% CI**	**95% CI**	***P***
**Treatment**								
UFT	1.037	0.954	1.126	0.397	0.964	0.891	1.041	0.349
5-FU	Reference				Reference			
**Gender**								
Male	1.380	1.266	1.504	<0.001	1.560	1.436	1.694	<0.001
Female	Reference				Reference			
**Age groups (yrs)**								
<30	Reference				Reference			
30-39	0.864	0.525	1.423	0.566	1.679	0.486	5.798	0.413
40-49	1.372	0.867	2.171	0.177	3.490	1.087	11.205	0.036
50-59	1.132	0.728	1.761	0.581	4.029	1.282	12.661	0.017
≧60	1.553	1.017	2.372	0.041	8.315	2.676	25.834	<0.001
**Insured premium (NT$)**								
<18,000	Reference				Reference			
18,000-34,999	0.663	0.443	0.991	0.045	0.617	0.409	0.930	0.021
≧35,000	0.634	0.238	1.693	0.363	0.383	0.096	1.532	0.175
**HTN**								
Without	Reference				Reference			
With	1.546	1.492	1.607	<0.001	1.312	1.278	1.350	<0.001
**DM**								
Without	Reference				Reference			
With	1.764	1.683	1.853	<0.001	1.787	1.712	1.872	<0.001
**COPD**								
Without	Reference				Reference			
With	1.785	1.666	1.925	0.004	1.068	0.944	1.209	0.297
**CKD**								
Without	Reference				Reference			
With	1.103	0.948	1.283	0.207	2.453	2.222	2.707	<0.001
**IHD**								

Without	Reference				Reference			
With	1.776	1.671	1.898	0.001	1.848	1.749	1.961	<0.001
**CHD**								
Without	Reference				Reference			
With	1.521	1.414	1.655	<0.001	1.631	1.447	1.839	<0.001
**Stroke**								
Without	Reference				Reference			
With	1.513	1.419	1.627	<0.001	1.097	0.957	1.257	0.184
**CCI_R**	1.142	1.079	1.209	<0.001	1.200	1.150	1.252	<0.001
**Urbanization level**								
1 (The highest)	0.971	0.844	1.117	0.680	1.173	1.030	1.335	0.016
2	1.062	0.936	1.204	0.352	1.219	1.086	1.370	0.001
3	0.855	0.702	1.040	0.117	0.889	0.741	1.068	0.209
4 (The lowest)	Reference				Reference			
**Level of care**								
Hospital center	1.060	0.933	1.204	0.373	0.666	0.595	0.744	<0.001
Regional hospital	0.946	0.843	1.062	0.349	0.694	0.628	0.766	<0.001
Local hospital	Reference				Reference			
**HR= hazard ratio, CI = confidence interval, Adjusted HR: Adjusted variables listed in the table**								
