# Supplementary material for: Uracil-tegafur vs fluorouracil as postoperative adjuvant chemotherapy in Stage II and III colon cancer: A nationwide cohort study and meta-analysis
Source: Medicine (Baltimore). 2021 May 7;100(18):e25756. doi: 10.1097/MD.0000000000025756 (PMC8104207; doi:10.1097/MD.0000000000025756)

Supplementary Digital Content 6. Flow diagram for the identification process for eligible studies

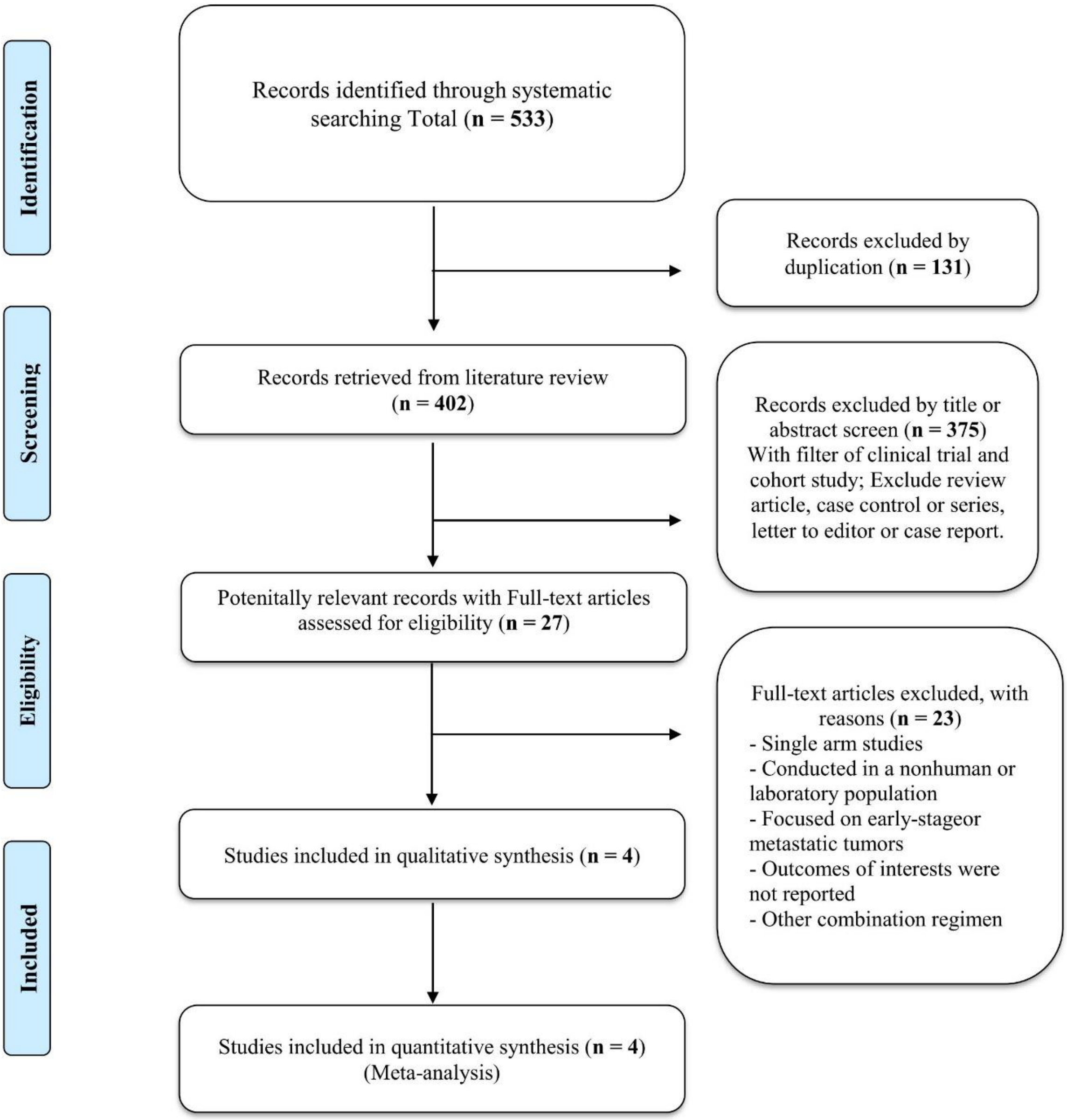

Supplement: Supplemental Digital Content [file medi-100-e25756-s007.pdf]
