# Supplementary material for: Uracil-tegafur vs fluorouracil as postoperative adjuvant chemotherapy in Stage II and III colon cancer: A nationwide cohort study and meta-analysis
Source: Medicine (Baltimore). 2021 May 7;100(18):e25756. doi: 10.1097/MD.0000000000025756 (PMC8104207; doi:10.1097/MD.0000000000025756)
Supplement: Supplemental Digital Content [file medi-100-e25756-s008.pdf]

Supplementary Digital Content 7. Basic characteristics of the included studies.

Study	Study Type	Region	Age (Mean)	Sex (Male, %)	Stage	Tumor Size (cm) Mean(SD)	Median Number of Positive Nodes	Histopathological classification	Number (Overall)
**Hu 2016**	Cohort	Taiwan	67.7/57.1	56.6%/54.8%	II: 100%/100%	Size: 5.1(2.5)/6.3(2.8)		Poorly differentiated: 8.6/9.6%	181
**Kim 2003**	Cohort	South Korea	56.2/55.7	62.3%/50.9%	II: 63.8%/64.2% III: 36.3%/35.8%	Size: 6.6(3.2)/6.2(2.2)		Poorly differentiated: 7.2/5.7%	122
**Shimada 2014 (JCOG0205)**	RCT	Japan	61/61 (Median)	54%/55%	IIIA: 15%/16% IIIB: 67%/66% IIIC: 18%/18%		2 (1–19)/2(1–37) (Median(Range))	Poorly differentiated: 7%/9%	1101
**Lembersky 2006 (NSABP C-06)**	RCT	USA	< 60 years: 41.2%/41.7% > 60 years: 58.8%/58.3%	53.5%51.6%	II: 46.7%/46.4% III: 53.3%/53.6%		1-3 positive nodes: 36.6%/38% > 4 positive nodes:16.3%/15.6%		1533

Study	Number (Arm 1/2)	Intervention 1	Intervention 2
**Hu 2016**	132/49	Oral UFT	IV bolus 5-FU
**Kim 2003**	69/53	300 mg/m2/day of oral UFT for 21 consecutive days followed by 7 days rest period of the 4-week course for 12 cycles	450 mg/m2 of IV bolus 5-FU on day 1, 8, 15 of the 4-week course for 12 cycles
**Shimada 2014 (JCOG0205)**	550/551	300 mg/m2/day of oral UFT for 4 weeks followed by 1 week rest period for five cycles	500 mg/m2 of IV bolus 5-FU weekly for 6 doses followed by 2 week rest period for three cycles
**Lembersky 2006 (NSABP C-06)**	774/79	300 mg/m2/day of oral UFT for 4 weeks followed by 1 week rest period for five cycles	500 mg/m2 of IV bolus 5-FU weekly for 6 doses followed by 2 week rest period for three cycles
