# Supplementary material for: Uracil-tegafur vs fluorouracil as postoperative adjuvant chemotherapy in Stage II and III colon cancer: A nationwide cohort study and meta-analysis
Source: Medicine (Baltimore). 2021 May 7;100(18):e25756. doi: 10.1097/MD.0000000000025756 (PMC8104207; doi:10.1097/MD.0000000000025756)
Supplement: Supplemental Digital Content [file medi-100-e25756-s009.pdf]

Supplementary Digital Content 8. Assessment of risk of bias and Newcastle-Ottawa Scale

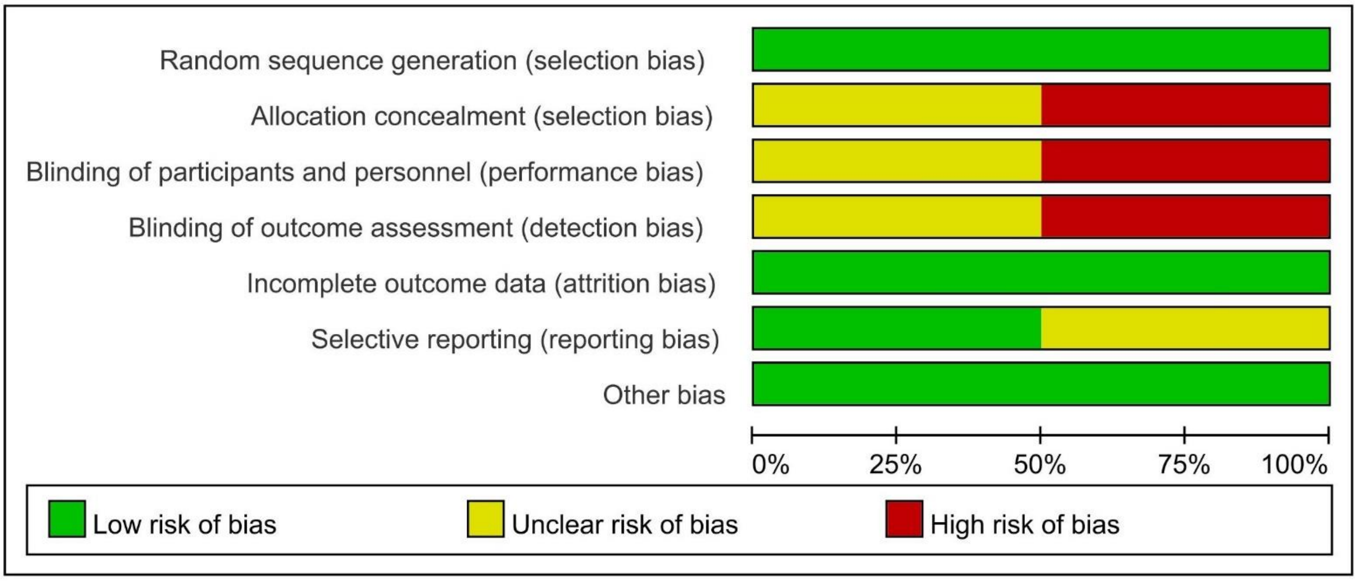

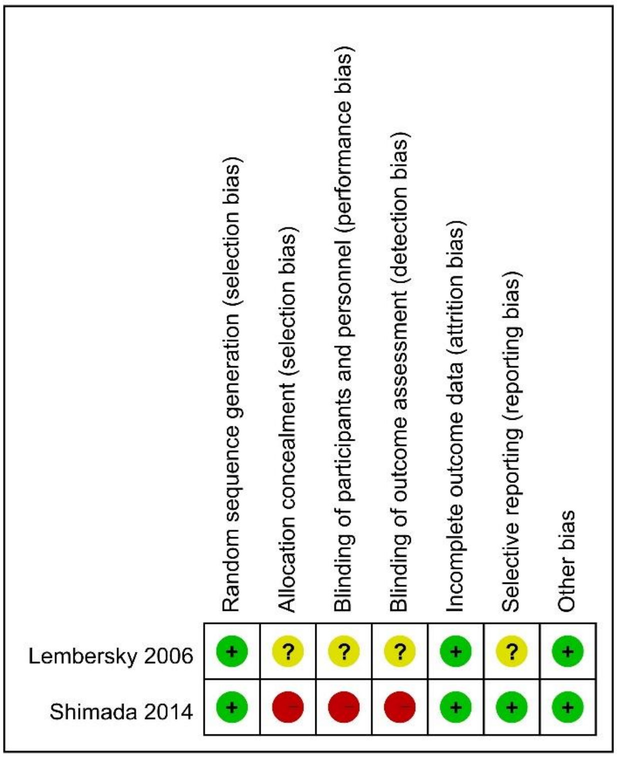

First author, year	Representativeness of the exposed cohort	Selection of the nonexposed cohort	Ascertainment of exposure	Demonstration that outcome of interest was not present at start of study	Comparability of cohorts on the basis of the design or analysis (2)	Assessment of outcome	Was follow-up long enough for outcomes to occur	Adequacy of follow up of cohorts
Hu 2016	*	*	*		*	*	*	*
Kim 2003	*	*	*		*	*		
Chen 2020	*	*	*	*	*	*	*	*
