# Supplementary material for: Uracil-tegafur vs fluorouracil as postoperative adjuvant chemotherapy in Stage II and III colon cancer: A nationwide cohort study and meta-analysis
Source: Medicine (Baltimore). 2021 May 7;100(18):e25756. doi: 10.1097/MD.0000000000025756 (PMC8104207; doi:10.1097/MD.0000000000025756)
Supplement: Supplemental Digital Content [file medi-100-e25756-s010.pdf]

Supplementary Digital Content 9. Subgroup analysis of outcomes

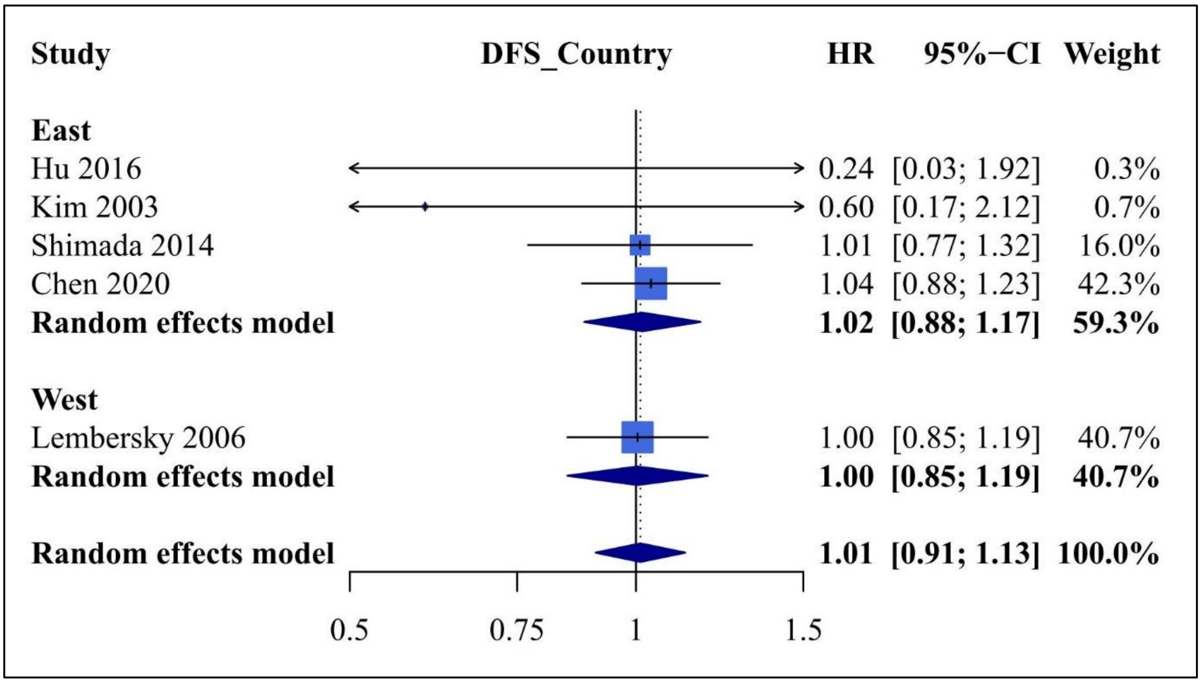

Subgroup analysis of study countries in outcome of disease-free survival

The included patients were categorized by performing studies in east or west countries. Outcome analyses were performed using hazard ratio (HR) with related 95% confidence intervals (95%CI). DFS, disease-free survival; HR, hazard ration; CI, confidence interval

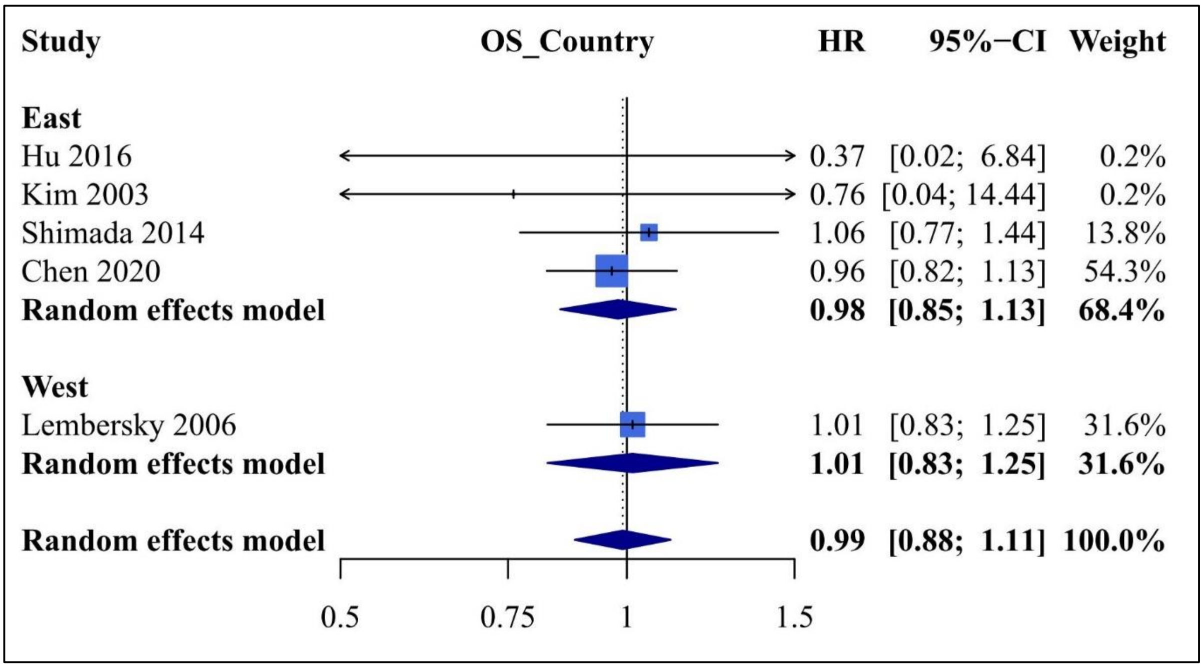

Subgroup analysis of study countries in outcome of overall survival

The included patients were categorized by performing studies in east or west countries. Outcome analyses were performed using hazard ratio (HR) with related 95% confidence intervals (95%CI).

OS, overall survival; HR, hazard ration; CI, confidence interval

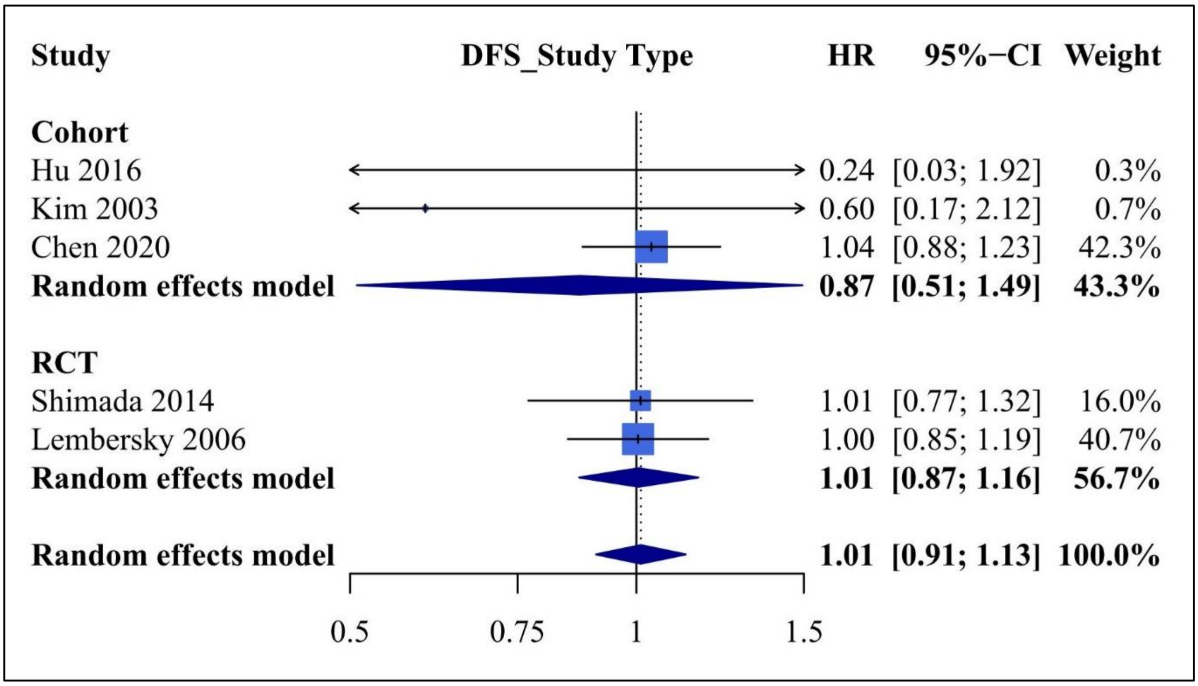

Subgroup analysis of study type in outcome of disease-free survival

The included patients were categorized by included studies types with cohort study or randomized control study. Outcome analyses were performed using hazard ratio (HR) with related 95% confidence intervals (95%CI).

DFS, disease-free survival; HR, hazard ration; CI, confidence interval; RCT, randomized control study

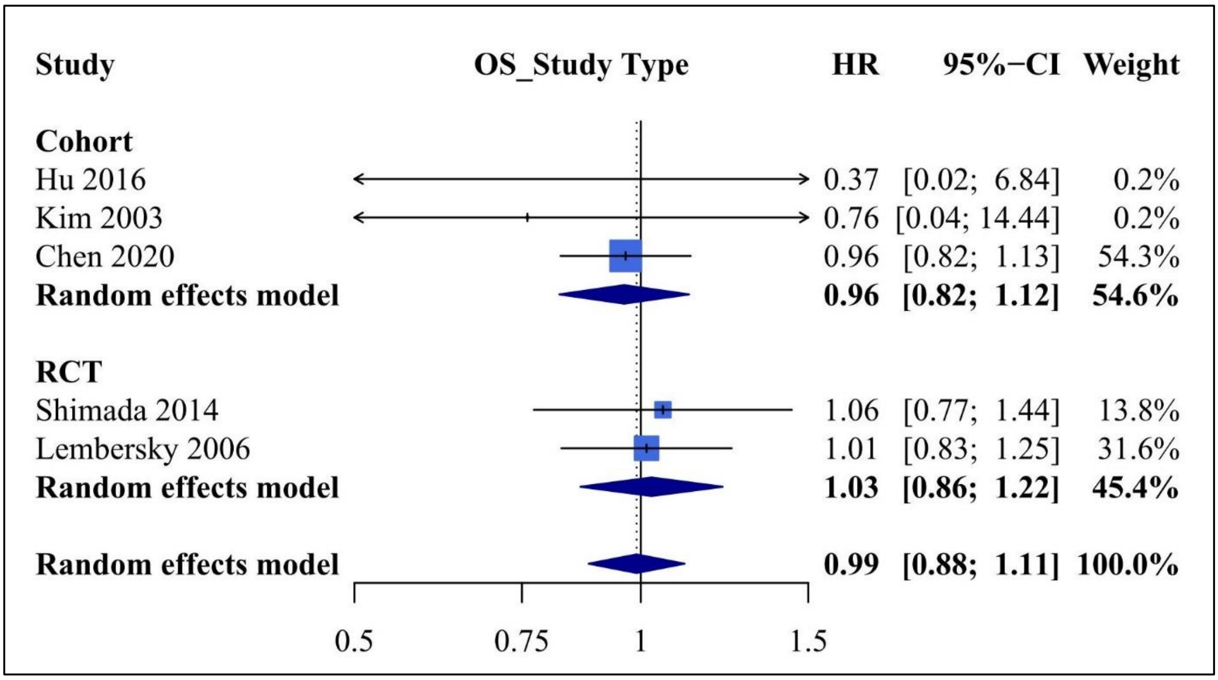

Subgroup analysis of study type in outcome of overall survival

The included patients were categorized by included studies types with cohort study or randomized control study. Outcome analyses were performed using hazard ratio (HR) with related 95% confidence intervals (95%CI).

OS, overall survival; HR, hazard ration; CI, confidence interval; RCT, randomized control study
